# Factors associated with the occurrence of *Triatoma sordida* (Hemiptera: Reduviidae) in rural localities of Central-West Brazil

**DOI:** 10.1590/0074-02760140395

**Published:** 2015-04

**Authors:** Juliana Chedid Nogared Rossi, Elisabeth C Duarte, Rodrigo Gurgel-Gonçalves

**Affiliations:** 1 Programa de Pós-Graduação em Medicina Tropical, Faculdade de Medicina; 2 Laboratório de Parasitologia Médica e Biologia de Vetores, Universidade de Brasília, Brasília, DF, Brasil

**Keywords:** Triatominae, Triatoma sordida, peridomicile, logistic regression, Chagas disease

## Abstract

This study estimates the factors of artificial environments (houses and peridomestic areas) associated with Triatoma sordida occurrence. Manual searches for triatomines were performed in 136 domiciliary units (DUs) in two rural localities of Central-West Brazil. For each DU, 32 structural, 23 biotic and 28 management variables were obtained. Multiple logistic regression analysis was performed in order to identify statistically significant variables associated with occurrence of T. sordida in the study areas. A total of 1,057 specimens (99% in peridomiciles, mainly chicken coops) of T. sordida were collected from 63 DUs (infestation: 47%; density: ~8 specimens/DU; crowding: ~17 specimens/infested DU; colonisation: 81%). Only six (0.6%) out of 945 specimens examined were infected with Trypanosoma cruzi. The final adjusted logistic regression model indicated that the probability of T. sordida occurrence was higher in DU with wooden chicken coops, presence of > 30 animals in wooden corrals, presence of wood piles and presence of food storeroom. The results show the persistence of T. sordida in peridomestic habitats in rural localities of Central-West Brazil. However, the observed low intradomestic colonisation and minimal triatomine infection rates indicate that T. sordida has low potential to sustain high rates of T. cruzi transmission to residents of these localities.

Chagas disease (CD) is endemic in rural populations inhabiting structurally deficient households that favour the colonisation of triatomine bugs. In 2006, the Pan American Health Organization declared that Brazil was free from *Trypanosoma cruzi* transmission by the domestic vector *Triatoma infestans* (Dias 2007 ). However, native species, such as *Panstrongylus megistus* (Villela et al. 2009), *Triatoma sordida* (Oliveira & Silva 2007), *Triatoma* *brasiliensis* and *Triatoma* *pseudomaculata* (Silva et al. 2012), continue to be found in domestic and peridomestic environments. Understanding the factors associated with household infestation by these species may identify new targets for intervention and minimise the risk of *T. cruzi* vectorial transmission (Cohen & Gürtler 2001).

House structure influences colonisation by triatomine bugs. Proximity of houses to vegetation and the presence of livestock both increase the likelihood of household infestation by triatomines. In addition, peridomestic structures may play an important role in maintaining triatomine populations in close proximity to residences (Campbell-Lendrum et al. 2007, Gurevitz et al. 2011, Dumonteil et al. 2013, Bustamante et al. 2014).

The factors associated with the occurrence of triatomine bugs were analysed in northeastern Brazil (Walter et al. 2005). However, these factors may vary in different regions of the country due to human behaviour, local vector ecology and socioeconomic conditions (Vinhaes et al. 2014). The identification of these factors in domestic and peridomestic environments is useful in order to prevent and control these bugs more effectively, thereby reducing the risk of vectorial transmission of CD (Weeks et al. 2013).

Traditionally, the most common synanthropic species captured in the Central-West Region of Brazil has been *T. sordida *(Pereira et al. 2013). This species occurs primarily in peridomestic environments, particularly chicken coops and therefore exhibit low rates of natural infection by *T.* *cruzi* (Forattini et al. 1975, Diotaiuti et al. 1995a, Oliveira & Silva 2007). Although several studies examining ecological and behavioural aspects of *T. sordida* have been published since the 1970s, the factors that determine its persistence in peridomiciliary environments need to be better understood. The relative importance of biotic, structural and environmental management factors influencing the occurrence of this species in Central-West Brazil has not been systematically evaluated; such research could result in more effective control procedures and lead to more reliable predictions of the likelihood of household infestation. The aim of this study therefore was to estimate the association between structural, biotic and environmental management factors and the presence of *T.* *sordida* in households in rural localities of the municipality of Posse, in the Brazilian state of Goiás (GO), Brazil.

## MATERIALS AND METHODS

*Study area* - The study was conducted in rural areas in Posse where CD is the main protozoan infection (Oliveira & Silva 2007). Data from the seroprevalence survey of *T. cruzi* human infection conducted between 1975-1980 showed an infection prevalence of 7.4% in the state, considered one of the highest rates in Brazil (Silveira et al. 2011). More recently, data collected from the Ministry of Health's Mortality Information System revealed that CD was responsible for 3,589 deaths in GO between 2007-2011. According to Martins-Melo et al. (2012), GO had the highest mortality rates resulting from CD in Brazil, mainly in the northeastern region of the state. Moreover, vector-borne acute cases of CD were confirmed in GO between 2006-2012, one of them in Posse (Vinhaes et al. 2014). However, the last attempt at vector-control in this municipality occurred in 2008.

**Fig 1A:**
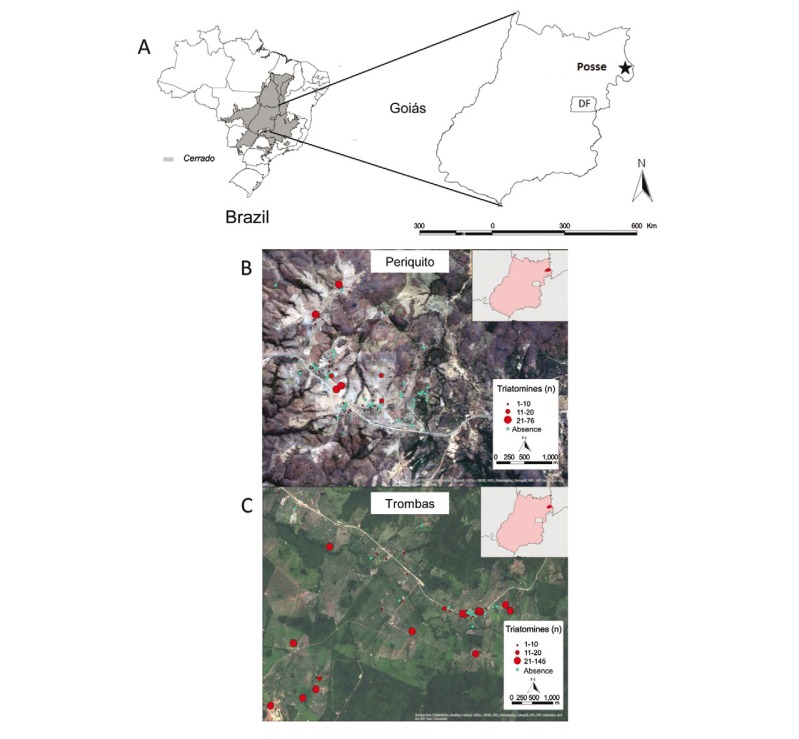
location of the municipality of Posse, state of Goiás, Brazil; B, C: Triatoma sordida distribution in the localities of Periquito and Trombas, respectively. The domiciliary units (DUs) are represented by circles (red: positive; green: negative). The size of the red circles represents the number of T. sordida specimens captured in the DUs: small (1-10), medium (11-20) and large (> 20 specimens).

The municipality of Posse is located in northeastern GO (Fig. 1A), approximately 320 km from Brasília, Federal District. It encompasses an area of 1,949.63 km^2^ and supports a population of approximately 31,257 individuals. This municipality is located in the *Cerrado* biome, where the climate is characterised by two well-defined seasons: the rainy season (October-March) and the dry season (April-September). High rates of triatomine infestation were observed in Posse in the 1980s and 1990s (Diotaiuti & Pinto 1991). Moreover, Posse was the last municipality in GO where *T. infestans* was recorded (Oliveira & Silva 2007) and systematic control programs have been inactive since 2008. Two localities within Posse (Trombas and Periquito) were selected because they had the highest infestation rates (11.6% and 5.5%, respectively) among all localities investigated in 2008.

*Triatomine collection and parasite detection* - The triatomine survey was conducted in April 2013. After resident permission was obtained, systematic manual triatomine searches in the domiciliary units (DUs) (the house itself plus any peridomiciliary annex buildings and the space between all such structures) were conducted by a team of two trained individuals equipped with gloves, flashlights and tweezers. The triatomine search required about 1 h per DU. Three visiting attempts on different days were performed when the resident was absent, after which the house was considered uninhabited or abandoned. The intradomicile inspection included all rooms in the DU. The walls, beds and other furniture, the spaces behind posters and frames, clothes baskets, accumulations of wood or bricks and any other sites that could represent a suitable refuge for insects were examined. The peridomiciliary environment was defined as the area surrounding the home, usually comprised of a fenced compound, regardless of the distance from the main house. In this environment, animal shelters (e.g., corrals, chicken coops and pig pens), bricks, wood, tiles and rocks were examined.

All DUs were georeferenced using a Garmin GPS 12 (GARMIN International^(r)^, USA) receiver. The spatial distribution of the DUs was determined after importing the geographical coordinates of each DU (latitude and longitude) into ArcGIS software (v.10.2). Positive and negative DUs in each location were then overlaid with satellite imagery.

Collected insects were stored alive in vials containing filter paper and properly labelled. The insects were separated by sex and nymphal stage and morphologically identified using the taxonomic keys described by Lent and Wygodzinsky (1979). Faeces of the collected triatomines were then examined for the presence of flagellates *via* direct microscopical observation. Natural infection was determined by examination of fresh faeces obtained by abdominal compression of the triatomine bugs. Parasites were morphologically identified by microscopical observation of Giemsa-stained insect faeces. Finally, standard entomological indicators (e.g., infestation, colonisation, density and crowding) (WHO 1991, Dias & Diotaiuti 1998) were estimated for each locality and habitat.

*Characterisation of DUs (houses and peridomestic area)* - For each DU, a form was filled out describing the features of the houses and of the peridomiciliary environment. The selection of the variables was based on previous studies analysing factors associated with triatomine bug infestation (Walter et al. 2005, 2007, Black et al. 2007, Campbell-Lendrum et al. 2007, Bustamante et al. 2009). In total, 32 structural, 23 biotic and 28 management variables (Table I) were included in our analysis.

**TABLE I.** List of structural, biotic and management factors of domiciliary units (DUs) from the localities of Trombas and Periquito, municipality of Posse, state of Goiás, Brazil, analysed in this study

Structural	Biotic	Management
Age of the house	Number of residents	DU was sprayed with insecticide
House wall	Time, in years, the house has been lived in	Time of last spraying
House roof	Presence of pets	DU received house improvement
House floor	Type of pets	Time since house improvement
Presence and place of the food storage	Animals sleeping indoors	Time, in years, of the house improvement
Number of rooms	Animals living indoors	Performs cleaning of the chicken coop
Number of windows	Number of domestic animals by type	Number of times the coop is cleaned in a month
Presence of electricity	Presence of a chicken coop	Distance, in meters, of the nearest chicken coop
Chicken coop wall	Number of chicken coops	Performs cleaning of the corral
Chicken coop roof	Number of chickens	Number of times the corral is cleaned in a month
Pig pen wall	Presence of corral	Distance, in meters, of the nearest corral
Pig pen roof	Number of pig pens	Performs cleaning of the pig pen
Corral wall	Number of animals in the corral	Number of times the pig pen is cleaned in a month
Corral roof	Presence of pig pen	Distance, in meters, of the nearest pig pen
Presence of barn	Number of pig pens	Performs cleaning of animal shelters^*a*^
Number of barns	Number of pigs	Number of times the animal shelters^*a*^ are cleaned in a month
Barn wall	Presence of animal shelter^*a*^	Distance, in meters, of animal shelters^*a*^
Barn roof	Number of animal shelters^*a*^	Performs cleaning of the barns
Presence and number of work tool storage^*b*^	Number of animals in these shelters	Number of times the barns are cleaned in a month
Work tool storage roof	Presence of loose peridomiciliary animals by type	Distance, in meters, of the nearest barn
Animal^*a*^ shelter wall	Presence of peridomiciliary palm trees	Performs cleaning of the work tool storage^*b*^
Animal shelter roof	Number of palm trees	Number of times the work tool storage^*b*^ is cleaned in a month
Presence of toilet outside the house	Presence of bird nests in the house	Distance, in meters, of the nearest work tool storage^*b*^
Number of toilets outside the house	-	Performs cleaning of the outside toilet
Toilet wall	-	Number of times the outside toilet is cleaned in a month
Toilet roof	-	Distance, in meters, of the nearest outside toilet
Presence and type of the fence	-	Lights on during the night
Presence of debris in the peridomicile by type	-	Distance to the nearest palm tree to the house

a : pets;

b : building to storage agriculture tools.

*Statistical analysis* - Descriptive statistics were estimated in relation to all DU variables. Crude and adjusted associations between explanatory variables and the dependent variable (presence or absence of *T. sordida* in DUs) were assessed using bivariated and multiple logistic regression models, using the PASW Statistics 18 program.

A final adjusted model based on multiple logistic regression was estimated using the backwards stepwise method for variable selection, as follows: (i) Pearson correlation matrix was used to identify collinearity among independent variables. Variables with high correlation coefficients (r ≥ 0.8) were considered collinear and only one was selected based on prior knowledge. (ii) Based on bivariated analysis (1 independent variable at a time), some variables were re-categorised to avoid collinearity and/or to improve statistical power by collapsing similar categories. (iii) Also based on bivariated analyses, independent variables whose association with *T. sordida* occurrence in DUs resulted in p-values < 0.20 were eligible for the multiple logistic regression analysis. (iv) Using the backwards variable selection approach, the least significant variables were excluded (1 by 1) from the model, until all variables (or some of its categories) were statistically associated with *T. sordida* occurrence at the p < 0.05 level.

## RESULTS

In total, 134 DUs were surveyed, composed of 70 (52.2%) in the locality of Trombas and 64 (47.8%) in the locality of Periquito. Some DUs were not surveyed because they were inhabited (24 in Trombas and 15 in Periquito) and/or closed (6 in Trombas and 2 in Periquito).

Most houses had full concrete walls (59%), ceramic tile (68%) and cement floors (84%). In the peridomiciles, the presence of wooden chicken coops (89%) and structures covered with asbestos tiles (46%) were very common. Most houses had a wire fence (52%) and peridomiciliary piles of material (85%) (e.g., wood, tile and bricks). The DUs features according to the variables related to structural, biotic and environmental management factors in each locality are present in Supplementary Tables I-III.

**TABLE II.** Entomological indicators for Triatoma sordida in the localities of Trombas and Periquito, municipality of Posse, state of Goiás, Brazil, 2013

Locality	Surveyed domiciliary units (n)	Infested domiciliary units (n)	Captured triatomines (n)	Infestation (%)	Colonisation (%)	Density	Crowding^*a*^
Trombas	70	39	783	55.7	76.9	11.2	20.1
Periquito	64	24	274	37.5	87.5	4.3	11.4
Total	134	63	1,057	47	81	7.9	16.8

a : number of captured triatomines/number of infested domiciliary units.

**TABLE III.** Numbers of captured, examined and infected specimens of Triatoma sordida by development stage in the localities of Trombas and Periquito, municipality of Posse, state of Goiás, Brazil, 2013

Locality/stages			
Trombas	Captured (n)	Examined (n)	Infected (n)
Nymph I	9	4	0
Nymph II	12	9	0
Nymph III	55	50	0
Nymph IV	86	77	1
Nymph V	241	229	4
Adult	380	329	1
Total	783	698	6
Periquito			
Nymph I	31	31	0
Nymph II	6	6	0
Nymph III	9	6	0
Nymph IV	14	14	0
Nymph V	37	35	0
Adult	177	155	0
Total	274	247	0

In total, 1,059 triatomine specimens were collected, consisting primarily of *T.* *sordida* (99%). Only two stage V nymphs of *Triatoma* *costalimai* were captured in the peridomiciliary environment. The infestation rate of DUs by *T.* *sordida* was 47%, while 81% of DUs were colonised by this species (Table II). Although adult and stage V nymphs of *T.* *sordida* predominated, specimens of all developmental stages were captured (Table III).

*T.* *sordida* was found to be widely distributed within the research areas (Fig. 1A, B). Periquito, closest to the urban centre of Posse, had a lower frequency of infested DUs, whereas the distribution of *T.* *sordida* in the localities showed that the DUs with the largest numbers of captured insects were located in Trombas (Fig. 1B), when compared to Periquito, where most of the DUs had densities lower than 20 individuals (Fig. 1C).

Only six specimens of *T.* *sordida* were infected with trypanosomatids in Trombas, corresponding to an infection rate of 0.6% (Table III). These trypanosomatids were morphologically similar to *T.* *cruzi.* The infected specimens were found in piles of tiles and in chicken coops.

In total, 692 ecotopes were surveyed, with chicken coops predominant (21.8%). The infestation rate by *T.* *sordida* was higher in chicken coops, where most of the insects were captured, resulting in higher density and crowding values. However, this species has been detected in other ecotopes, including barns, tool storage sheds, corrals, pigpens and other animal-shelter structures. The colonisation rate of *T.* *sordida* was higher in corrals and barns. Only four uninfected adult *T. sordida* were captured inside houses (Table IV).

**TABLE IV.** Entomological indicators for Triatoma sordida by ecotope in the localities surveyed, municipality of Posse, state of Goiás, Brazil, 2013

Ecotopes	Surveyed (n)	Infestation (%)	Colonisation (%)	Captured specimens (n)	Density	Crowding
Chicken coops	151	37.1	60.7	705	4.7	12.6
Corral	37	2.7	100	6	0.2	6.0
Pigsties	73	12.3	66.7	34	0.5	3.8
Pet shelters	22	9.1	50	3	0.1	1.5
Barn	51	23.5	91.7	114	2.2	9.5
Work tool storage	49	6.1	66.7	4	0.1	1.3
Peridomestic bathroom	57	0	-	0	0.0	-
Food storeroom	9	0	-	0	0.0	-
Fence	109	0	-	0	0.0	-
Intradomiciles	134	2.2	0	4	0.03	1.3
Piles^*a*^	-	-	-	187	-	-

a : wood, tile, brick and garbage.

These ecotypes were not quantified; we only detected the presence and absence of them in the domiciliary units.

The occurrence of *T.* *sordida* in DUs was associated with the presence of animals (cats, dogs and chickens), brick, wood, tile and rock piles surrounding the house, structure of the chicken coops and management variables such as distance of chicken coop from the house and cleanliness of peridomestic annexes (Supplementary Tables IV-VI).

**TABLE V.** Adjusted odds ratios (OR) and its 95% confidence intervals (CI) estimated by multiple logistic regression (final model) for the associations between selected exposure variables and infestation by Triatoma sordida of domiciliary units, municipality of Posse, state of Goiás, Brazil, 2013

	Adjusted OR	95% CI		
Variables		Inferior	Superior	p
Locality				
Periquito	1.00	-	-	-
Trombas	4.04	1.595	10.250	0.003
Chicken coop structure				
Chicken coop absence	1.00	-	-	-
Other structure	9.75	0.483	196.689	0.137
Brick	3.04	0.245	37.769	0.386
Mixed (wood + brick)	22.59	0.996	512.484	0.050
Wood	7.45	1.381	40.189	0.020
Animals in the corral				
Corral absence	1.00	-	-	-
Corral with up to 9 animals	2.73	0.569	13.107	0.209
Corral with 10-26 animals	3.20	0.529	19.379	0.205
Corral with > 30 animals	7.15	1.120	45.696	0.038
Peridomicile wooden piles				
Wooden pile absence	1.00	-	-	-
Presence of other piles	5.10	0.797	32.634	0.085
Presence of wooden piles	8.53	1.541	47.216	0.014
Food storeroom				
No	1.00	-	-	-
Yes, inside house	3.458	1.323	9.040	0.011
Yes, outside house	7.178	1.022	50.415	0.047

**Fig 2:**
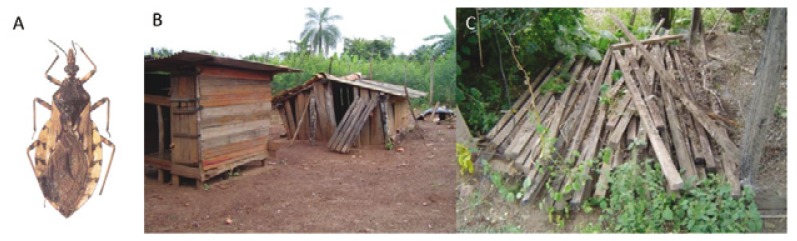
peridomestic ecotopes for Triatoma sordida (A). Wooden chicken coops (B) and wooden piles (C) presented in the peridomestic area of localities in the municipality of Posse, state of Goiás, Brazil.

Among the 34 variables selected to be included in the multiple logistic regression model, five remained statistically associated with *T.* *sordida* occurrence in the final model (Table V). The results indicate that the probability of occurrence of *T.* *sordida* is four times higher in Trombas and, in DUs with wooden chicken coops (Fig. 2A, B), corrals holding > 30 animals, DUs with wooden piles in the peridomestic area (Fig. 2C) and those with food storage structures.

## DISCUSSION

This study estimated factors associated with *T.* *sordida* occurrence in rural localities in the Central-West Region of Brazil. The results showed that factors related to structural characteristics (e.g., wooden chicken coops and pens, wood piles around houses and food storages) and biotic (e.g., number of animals in a corral) are the factors that best explain the occurrence of this triatomine species.

The results obtained in this study revealed high peridomiciliary infestation rates by *T.* *sordida*, especially in chicken coops. Furthermore, no nymphs were captured inside the houses, indicating a small likelihood of house colonisation by *T.* *sordida* in the study areas. The dominance of this species in peridomiciles associated with the low rate of natural infection are in agreement with results of other studies conducted in GO (Oliveira & Silva 2007) and in the states of Minas Gerais (MG) (Diotaiuti et al. 1995a), São Paulo (Forattini et al. 1975) and Bahia (BA)(Pires et al. 1999). Historically, *T. sordida* has been the most frequently captured triatomine species in Brazil (Diotaiuti et al. 1998, Silveira & Dias 2011). Even in some areas where there was a predominance of *T.* *infestans* in the 1970s (e.g., north of MG), after chemical control, *T.* *sordida* became the predominant species following control programs for *T. infestans* (Diotaiuti et al. 1995a). The high rate of occurrence of *T. sordida* in chicken coops is consistent with the known ornithophilic habits of this species and to the high availability of this food source in rural areas of Brazil (Forattini et al. 1982, Pires et al. 1999).

Only two nymphs of *T.* *costalimai* were collected during field research, with one specimen being found in a chicken coop and the other in a tool storage shed. Despite the infrequency of this species in this study, the identification of these nymphs may indicate colonisation of these ecotopes. It is important to note that the DU where the nymphs were collected was located near rock outcroppings, common habitats for this species (Mello 1981). *T. costalimai* has also been found in the peridomiciliary areas of other municipalities in GO (Oliveira & Silva 2007, Machiner et al. 2012).

Dispersion of *T.* *sordida* can occur both passively (e.g., by transporting firewood to domiciles or by birds carrying nymphs during flight) and actively by flying adults (Forattini et al. 1971). According to Forattini et al. (1975), *T. sordida* is highly active compared to other triatomine species. These ecological studies, coupled with feeding and defecation dynamics studies (Diotaiuti et al. 1995b), suggested that *T. sordida* would play an important role in the transmission of *T.* *cruzi.* Noireau et al. (1997), on the other hand, demonstrated that the probability of *T. cruzi* transmission to humans by domiciliary *T. sordida* in Bolivia was low. Moreover, vectorial transmission of *T. cruzi* was strongly reduced after *T. infestans* control measures were implemented in Brazil, even with the permanence of *T.* *sordida* in rural areas (Silveira & Dias 2011). However, *T. sordida* can maintain *T. cruzi* cycles in peridomestic environments (Diotaiuti et al. 1995c). *T. sordida* was implicated as a potential vector of *T. cruzi* to humans in an oral outbreak of acute DC in BA. A colony of *T. sordida* was detected in the kitchen area of the house, where residents stored food; 50% of the triatomines were positive for *T. cruzi* and precipitin tests indicated association with birds, opossums, rodents and humans. Additionally, an entomological survey performed around the house revealed that 40% of the *T. sordida *specimens collected were infected with *T. cruzi* (Dias et al. 2008). These results indicate the importance of maintaining monitoring and control programs in areas infested with *T. sordida*.

Structures made of wood predominated in the peridomiciles in the study area. The results of the logistic regression showed that the presence of a wooden chicken coop adjacent to the house is a factor associated with *T.* *sordida* occurrence in the studied areas. One possible explanation is that, in the wild, this species is often found under tree bark and in hollow trees of the *Cerrado* biome (Forattini et al. 1971, Diotaiuti et al. 1993).

The presence of wood piles as an associated factor for the occurrence of other triatomine species, such as *T.* *pseudomaculata* (Walter et al. 2005), *Triatoma* *pallidipennis* (Cohen et al. 2006) and *Triatoma* *longipennis* (Walter et al. 2007), has also been described, indicating the importance of proper environmental management of these breeding ecotopes for triatomine control. The association between storerooms and occurrence of *Rhodnius* *prolixus* (Campbell-Lendrum et al. 2007), *T.* *infestans* (Gurevitz et al. 2011) and *Triatoma dimidiata* (Bustamante et al. 2014) has also been recorded, suggesting that storerooms provide additional places for triatomine refuge. Food storage also provides resources for other animals, such as synanthropic rodents, which could act as blood sources for triatomines. In Brazil, the association between triatomines and corrals had been described for *T.* *pseudomaculata* in BA (Walter et al. 2005, Resgo et al. 2006).

Our results suggest that changes in the construction of chicken coops may decrease the occurrence of *T. sordida.* Improving peridomestic structures, removal of piles of material and cleaning the peridomicile also reduce food sources and hiding places for triatomines (Lucero et al. 2013). The present study recommends the replacement of wood with wire for chicken coop construction and increasing the distance between houses and the coops, in addition to more frequent cleaning of coops. Longitudinal studies that measure the rate of infestation of chicken coops before and after these actions would be useful for examining the effectiveness of this type of environmental management. Studies have shown that not only is environmental management effective in maintaining low triatomine infestation, but it also improves the effectiveness of insecticide spraying because it reduces the attractiveness of such ecotopes as sites for triatomine reproduction (Gorla et al. 2013, Lucero et al. 2013, Stevens et al. 2013). The improvement of chicken coops appears to be a promising strategy, considering the difficulties of controlling *T.* *sordida* in peridomestic environments using insecticides. According to Diotaiuti et al. (1998), spraying does not completely eliminate the bugs due to the low residual activity of the insecticide in peridomiciles.

Although the structural, biotic and environmental management features were similar between the two localities studied (Supplementary Tables), other variables that were not sampled could explain the differences between the entomological indicators of these locations. The higher infestation rates observed in Trombas could be a result of its greater distance from the administrative centre of Posse (which could hinder control activities) because it contains the lowest proportion of households that have undergone housing improvements (Supplementary Tables) and because of the history of land use in these locations (e.g., deforestation and agriculture). Insecticide control performed by residents of Periquito may also explain the differences in DU infestation between the two locales. Future studies that include more locations could better assess differences in the occurrence of *T. sordida* and expand the inferences obtained in this study.

Limitations of the present study included the relatively small number of localities sampled, the high number of DUs that were not sampled (47 houses were closed or uninhabited) and the method of sample collection. Manual searching is the standard method used for the detection of triatomine bugs, but its limited sensitivity can often result in underestimations of the values of entomological indicators and detection error/bias may occur at low bug densities. Repeated surveys using baited traps may increase the detection sensitivity within houses (de Arias et al. 2012, Abad-Franch et al. 2014).

The results of this study suggest that *T. sordida* has low potential for sustaining high rates of *T. cruzi* transmission to residents of the studied areas, as in other areas with rural characteristics similar to those in Trombas and Periquito. However, due to the high infestation rates of the peridomestic ecotopes and to the observed associated factors, we recommend that routine and efficient entomological monitoring programs that are capable of detecting changes in the behaviour of the species be maintained, in order to identify any incipient household colonisation by *T.* *sordida* or other native species.
